# Mobilizing Progress: A Comprehensive Review of the Efficacy of Early Mobilization Therapy in the Intensive Care Unit

**DOI:** 10.7759/cureus.57595

**Published:** 2024-04-04

**Authors:** Amol Singam

**Affiliations:** 1 Critical Care Medicine, Jawaharlal Nehru Medical College, Datta Meghe Institute of Higher Education & Research, Wardha, IND

**Keywords:** rehabilitation, immobility, functional outcomes, critically ill patients, intensive care unit (icu), early mobilization therapy

## Abstract

Early mobilization therapy has emerged as a crucial aspect of intensive care unit (ICU) management, aiming to counteract the detrimental effects of prolonged immobility in critically ill patients. This comprehensive review examines the efficacy of early mobilization therapy in the ICU setting, synthesizing evidence from clinical trials, meta-analyses, and guidelines. Key findings indicate that early mobilization is associated with numerous benefits, including reduced muscle weakness, a shorter duration of mechanical ventilation, decreased ICU and hospital length of stay, and improved functional outcomes. However, safety concerns, staffing limitations, and patient-specific considerations pose significant barriers to widespread adoption. Despite these challenges, early mobilization is important for improving ICU patient outcomes. This review underscores the critical need for continued research and implementation efforts to optimize early mobilization protocols, address remaining challenges, and expand access to this beneficial therapy. By working collaboratively to overcome barriers and prioritize early mobilization, healthcare providers can enhance the quality of care and improve outcomes for critically ill patients in the ICU.

## Introduction and background

Early mobilization therapy is a systematic approach to initiating physical activity and movement in critically ill patients in the intensive care unit (ICU) [1]. Traditionally, ICU patients are often kept sedated and immobilized to prevent complications and facilitate medical management. However, research over the past few decades has highlighted the detrimental effects of prolonged immobility, such as muscle weakness, ventilator-associated complications, and psychological distress. Early mobilization therapy aims to counteract these negative consequences by promoting early and progressive physical activity tailored to the patient’s condition and capabilities [2].

The significance of early mobilization for ICU patients cannot be overstated. Prolonged immobility in the ICU has been associated with a myriad of adverse outcomes, including muscle atrophy, weakness, ventilator-associated pneumonia (VAP), pressure ulcers, and an increased risk of thromboembolic events [3]. Moreover, immobility can exacerbate psychological distress, leading to anxiety, depression, and post-traumatic stress disorder (PTSD) in ICU survivors. By contrast, early mobilization has been shown to mitigate these risks, improve functional outcomes, shorten ICU and hospital lengths of stay, and enhance survivors’ overall quality of life [4].

This review aims to comprehensively examine the efficacy of early mobilization therapy in the ICU setting. By synthesizing the existing literature, we aim to elucidate the benefits, challenges, and implications of early mobilization for critically ill patients. Furthermore, we seek to identify gaps in knowledge, areas for future research, and strategies for successfully implementing early mobilization protocols in the ICU. Ultimately, this review aims to contribute to optimizing ICU care and improving outcomes for critically ill patients by promoting early mobilization therapy.

## Review

Background

Historical Perspective on ICU Care and Immobility

The historical perspective on ICU care and immobility delineates the progression of critical care medicine and nursing practices over time. In the 19th century, professional nurses delivered physical care in hospitals near nursing stations, marking the nascent stages of intensive therapy. The advent of life-support devices for ventilation and renal function characterized early 20th-century intensive therapy. Over the past five decades, critical care has evolved into comprehensive monitoring and automated laboratory measurements guided by critical care physicians, nurse specialists, pharmacists, and respiratory therapists utilizing diverse life-support methodologies [5]. During the 1990s, a significant paradigm shift unfolded in ICU critical care medicine toward managing more critically ill patients with conditions like acute respiratory distress syndrome. During this period, many breakthroughs occurred in sustaining patients on ventilators for extended durations and exploring novel treatment modalities. However, practices involving deep sedation and paralysis were prevalent, resulting in prolonged immobility and potential long-term ramifications for patients [6]. The ICU Liberation initiative by the Society of Critical Care Medicine is geared toward saving patients from pain, oversedation, delirium, immobility, and sleep disturbances in the ICU. Implementing strategies such as ventilator weaning protocols, maintaining light sedation levels, preventing delirium, initiating early mobilization, and fostering family engagement aims to enhance patient outcomes and reduce the risk of post-intensive care syndrome (PICS) [7]. In recent years, critical care nursing has witnessed advancements in multidisciplinary teamwork, protocol-driven care for weaning from mechanical ventilation and sedation, early patient mobilization to prevent complications like VAP and deep vein thrombosis, as well as a heightened emphasis on humanizing ICU environments through open visiting policies and ethical end-of-life care approaches [8].

Evolution of Early Mobilization Therapy

Over the past decade, the evolution of early mobilization therapy in the ICU has marked significant progress. Numerous randomized trials have been conducted to assess the efficacy of early mobilization and rehabilitation in ICU settings, reduce the incidence of ICU-acquired weakness (ICUAW), and enhance long-term physical functioning and quality of life for patients. Despite the availability of supportive evidence and guidelines advocating for early mobilization, its implementation in ICUs exhibits considerable variability [9]. Recent studies have shed light on various strategies to optimize ICU early mobilization and rehabilitation practices. These strategies encompass the establishment of multidisciplinary teams (MDTs) with assigned champions, the utilization of structured quality improvement methodologies, the identification of barriers and facilitators, the assessment of optimal timing, type, and dosage of interventions, the evaluation of outcomes and performance metrics, and the integration of mobility-related measures into clinical care to establish patient-centric goals and monitor progress [10]. The effectiveness of early mobilization in the ICU has been corroborated by research demonstrating a reduction in the incidence of ICUAW, enhancement of functional capacity, reduction in mechanical ventilation duration, improvement in patient’s ability to stand, increased rates of ICU discharge, and overall improvement in patient outcomes. Emerging techniques such as electrical muscle stimulation, cycling, hydrotherapy, and devices like the Sara Combilizer have exhibited favorable outcomes and safety profiles in facilitating early mobilization [3].

Physiological and psychological effects of immobility in ICU patients

Muscle Atrophy and Weakness

Muscle atrophy and weakness represent significant challenges encountered by critically ill patients in the ICU. ICUAW is a prevalent condition among critically ill individuals, affecting up to 80% of patients. It can result in prolonged disability extending far beyond the ICU stay. ICUAW manifests as muscle wasting, compromised contractility, neuropathy, and dysregulation of pathways involved in muscle protein degradation, such as the ubiquitin-proteasome system [11]. Notably, the preferential loss of myosin is a distinguishing characteristic of this condition. Risk factors contributing to ICUAW include inflammation, the administration of steroids, and immobilization through paralysis, underscoring the critical importance of early rehabilitation interventions in mitigating these declines in muscle function [11]. Research indicates that daily rates of muscle atrophy in key muscle groups such as the rectus femoris and vastus intermedius can be alarmingly high, reaching up to 0.84% and 0.98%, respectively. Furthermore, gender disparities do exist, with women experiencing approximately three times higher rates of muscle atrophy compared to men [12]. A comprehensive understanding of the mechanisms underlying muscle atrophy and weakness in critically ill patients is imperative for developing effective interventions to prevent and manage these conditions. Ultimately, such interventions promise to improve patient outcomes and significantly enhance overall quality of life.

Ventilator-Associated Complications

Ventilator-associated complications, including VAP, ventilator-associated events (VAEs), and infection-related ventilator-associated complications (IVACs), pose significant challenges for patients in the ICU. Research indicates that VAP is a prevalent ICU-acquired infection, often correlated with prolonged mechanical ventilation, extended stays in the ICU and hospital, and elevated mortality rates. Reported incidences vary widely, from 5% to 40%, depending on the setting and diagnostic criteria. VAP is associated with prolonged mechanical ventilation and ICU stays [13,14]. VAEs, encompassing both VAC and IVAC, have been identified as contributors to adverse patient outcomes, with IVAC linked explicitly to increased hospital mortality among critically ill patients necessitating prolonged mechanical ventilation [15,16]. These complications underscore the critical need for vigilant surveillance and the implementation of preventive strategies to reduce ventilator-associated complications’ impact on patient outcomes within the ICU.

Psychological Impact of Prolonged Immobility

Prolonged immobility in ICU patients can take a profound toll on their psychological well-being, precipitating conditions such as depression, anxiety, cognitive impairments, and PTSD. Research indicates that as many as 80% of critically ill patients in the ICU develop neuromuscular dysfunction or ICU delirium, both of which are closely linked to unfavorable outcomes [17]. Moreover, survivors of critical illness often grapple with moderate to severe depression and anxiety persisting for up to two years post-discharge, with depression rates surpassing those observed in the general population [17]. Cognitive impairments, although frequently overlooked, can persist over the long term, significantly impacting the quality of life for ICU survivors [17]. Furthermore, the psychological ramifications following critical illness, collectively referred to as PICS, encompass a spectrum of physical, mental, and cognitive challenges. Depression and symptoms of PTSD constitute pivotal components of psychological PICS and are pivotal factors associated with patient-reported unacceptable outcomes [18]. These psychological sequelae have been documented to endure for up to five years post-ICU discharge, underscoring the imperative of addressing and managing these psychological burdens to bolster the quality of life for ICU survivors [18].

Components of early mobilization therapy

Bedside Exercises

The components of early mobilization therapy, particularly bedside exercises, encompass a spectrum of interventions meticulously tailored to the patient’s individual tolerance levels and requirements. These interventions range from straightforward activities, such as transitioning from a supine to a seated position or performing grooming tasks while seated at the bedside, to more advanced maneuvers, like ambulating to the bathroom. The intervention selection should be driven by a patient-centered approach, considering their capacity to withstand and engage in the activity [19]. In instances where patients exhibit diminished endurance or strength, co-treatment involving collaboration with physical therapy can prove advantageous. This collaborative approach facilitates patient participation in early mobilization activities with appropriate assistance, thereby fostering favorable outcomes such as reduced length of stay and enhanced functional recovery [19]. Crucially, vigilant monitoring of patients’ vital signs throughout the mobilization session is imperative to ensure their tolerability of the intervention and to promptly document any observed changes for subsequent treatments [19]. While certain patients may not be deemed suitable candidates for early mobilization due to medical instability or other complicating factors, a significant portion of patients stand to benefit from these interventions with minimal adverse effects, underscoring the pivotal role of early mobilization in ameliorating patient outcomes in both ICU and acute care settings [19,20].

Sitting on the Edge of the Bed

Early mobilization therapy encompasses a variety of components designed to safely mobilize critically ill patients within the ICU. One crucial element involves transitioning patients from prone positions to sitting on the edge of the bed or in a chair [19,21]. Additionally, early mobilization interventions may encompass basic grooming tasks. At the same time, the patient is seated at the edge of the bed, transferring to a bedside commode, ambulating to the bathroom, or engaging in activities of daily living (ADLs) retraining tailored to the patient’s tolerance and requirements [19]. When patients exhibit diminished endurance or strength, concurrent treatment with physical therapy can facilitate early mobilization activities and ultimately improve outcomes [19]. It is essential to monitor patients’ vital signs before, during, and after each mobilization session to ensure they are tolerating the intervention well and to track their progress effectively [19]. While not all patients may be deemed suitable candidates for early mobilization due to medical instability or other complicating factors, integrating these components into therapy protocols can help reduce the risk of functional decline and enhance outcomes for many critically ill patients in the ICU.

Ambulation and Walking

The components of early mobilization therapy, with a particular focus on ambulation and walking, encompass various techniques to enhance functional outcomes for critically ill patients in the ICU. These components comprise a spectrum of activities, including passive and active range of motion exercises, active side-to-side turning, bed exercises, bedside sitting, transfers between bed and chair, ambulation, hoist therapy, tilt table exercises, resistance exercises, and electrical stimulation [1]. Early mobilization entails initiating physical activity as soon as the second to fifth day following the onset of a critical illness, underscoring the critical importance of mobilizing patients promptly to reduce complications associated with prolonged immobility [21]. Research indicates that early rehabilitation and mobilization in the ICU yield notable improvements in functional capacity, muscle strength, duration of mechanical ventilation, walking ability upon discharge, and health-related quality of life [21]. These interventions play a pivotal role in reducing the incidence of ICUAW, increasing ventilator-free days, and heightening the likelihood of patients being discharged home [21].

Physical Therapy Techniques

**Figure 1:**
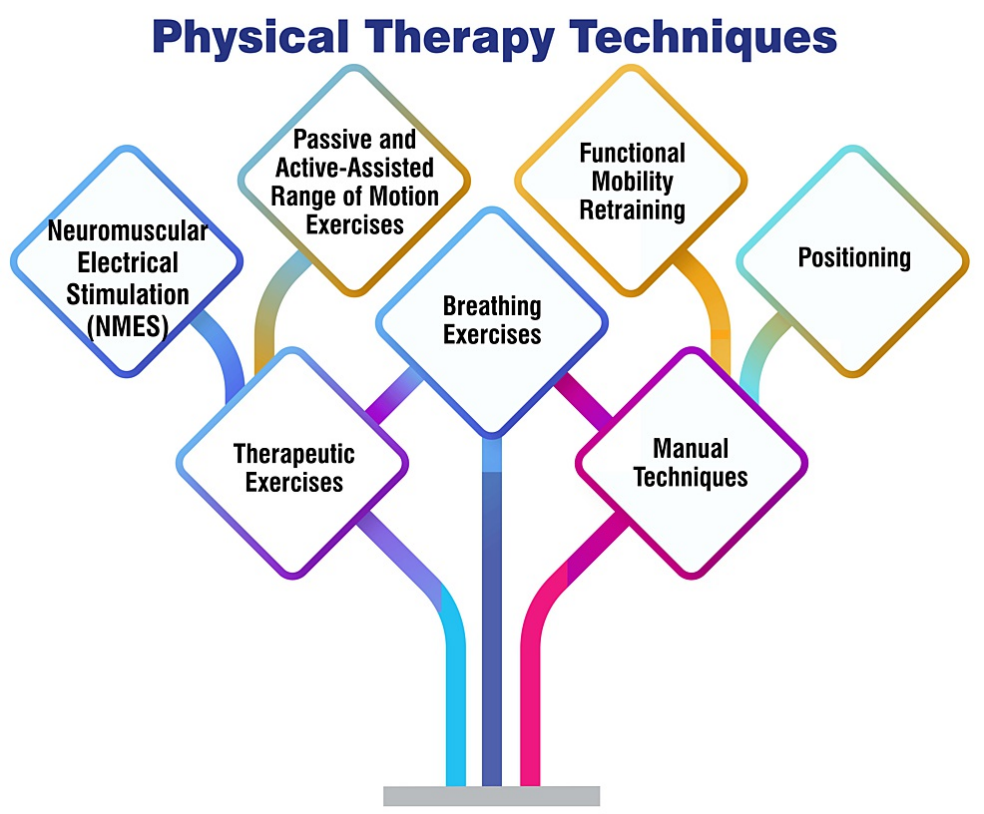
Physical therapy techniques Image credit: Amol Singam

Passive and active-assisted range of motion exercises involve manipulating the patient’s joints through their full range of motion, either with assistance (active assisted) or without requiring the patient’s effort (passive). These exercises are instrumental in averting joint stiffness and preserving flexibility [22]. Functional mobility retraining is a targeted technique to enhance a patient’s capacity to engage in daily activities and move autonomously. This approach incorporates exercises that replicate functional tasks such as sitting, standing, and walking, thereby facilitating the restoration of functional independence [23]. Positioning is paramount in the ICU setting to forestall complications such as pressure ulcers, contractures, and respiratory issues. Physical therapists are pivotal in ensuring optimal patient positioning to promote comfort and safety [22]. Breathing exercises are relevant for ICU patients, particularly those reliant on mechanical ventilation. These exercises facilitate lung function, reduce respiratory complications, and bolster overall respiratory health [22]. Therapeutic exercises encompass a diverse range of movements customized to meet the patient’s specific needs, targeting areas such as strength, endurance, balance, and coordination. These exercises enhance physical function and overall well-being and contribute to the patient’s rehabilitation [23]. Neuromuscular Electrical Stimulation involves the application of electrical stimulation to activate muscles, aiding in muscle strengthening and averting muscle atrophy in critically ill patients [24]. Manual techniques employed by physiotherapists encompass methods such as percussion, vibration, and manual hyperventilation to assist patients in clearing secretions, improving lung function, and enhancing overall respiratory health [25]. Figure 1 shows physical therapy techniques.

Evidence supporting early mobilization in ICU patients

Clinical Trials and Studies Demonstrating Benefits

Clinical trials and studies have unequivocally demonstrated the advantages of early mobilization within the ICU. Seminal studies have conclusively shown that initiating early mobilization can reduce ICU and hospital stay durations, hasten return to independence, enhance ambulation capacity, augment muscle strength, reduce ICUAW, and amplify functional recovery [26,27]. Research further underscores that early active mobilization during mechanical ventilation in the ICU contributes to shortened ICU stays and heightened functional mobility [27]. Moreover, systematic reviews have illuminated that early physical therapy and ICU mobilization represent feasible, safe, and productive interventions. These interventions facilitate the attainment of mobility milestones and yield tangible improvements in functional outcomes for critically ill patients within the ICU [28].

Meta-Analyses and Systematic Reviews

The meta-analyses and systematic reviews examine the effects of early mobilization therapy on critically ill patients within the ICU. These comprehensive studies scrutinize the influence of early mobilization on diverse outcomes, including muscle strength, physical function, duration of ICU and hospital stays, and the occurrence of adverse events among patients undergoing cardiac surgery or requiring mechanical ventilation [10,29-32]. The cumulative evidence suggests that early mobilization holds promise in enhancing physical function, mitigating the risk of ICUAW, expediting the weaning process from mechanical ventilation, and potentially forestalling ICUAW, a condition associated with diminished quality of life and heightened mortality risk [10]. Nevertheless, the certainty of the evidence regarding the benefits of systematic early mobilization remains inconclusive. Some studies have reported conflicting findings, underscoring the imperative for further research to elucidate the effectiveness of early mobilization interventions in the ICU [30,31]. Despite these uncertainties, meta-analyses and systematic reviews underscore early mobilization’s significance as a prospective intervention to facilitate outcomes for critically ill patients. However, additional research is warranted to grasp its impact comprehensively.

Guidelines and Recommendations from Professional Organizations

Guidelines and recommendations from professional organizations underscore the critical importance of early mobilization in the ICU to enhance patient outcomes. These guidelines advocate for implementing early mobility protocols to foster patient mobility and facilitate functional recovery within the ICU setting [33,34]. Extensive research has demonstrated that early mobilization is feasible, safe, and effective in reducing the length of ICU stays, improving functional outcomes, and mitigating complications associated with prolonged immobilization [33,35]. The evidence supports the utilization of interdisciplinary collaboration, education, and targeted interventions to optimize the feasibility and effectiveness of early mobilization programs in the ICU [33,35]. Additionally, the literature underscores the manifold benefits of early ambulation as an integral component of a comprehensive care bundle within the ICU, leading to tangible improvements such as decreased mechanical ventilation duration, abbreviated ICU and hospital stays, and enhanced functional recovery for critically ill patients [33]. Despite potential barriers such as resource allocation constraints and apprehensions regarding patient safety, protocols and guideline recommendations have been developed to address these concerns and ensure the safety and appropriateness of early mobilization care in the ICU [33,35]. In summary, professional guidelines and recommendations underscore early mobilization’s pivotal role in augmenting outcomes and mitigating complications for critically ill patients within the ICU setting.

Challenges and considerations

Safety Concerns and Risk Assessment

Safety concerns and risk assessment represent crucial considerations when contemplating early mobilization therapy within the ICU. Studies underscore the importance of establishing safety criteria for initiating early mobilization, particularly for patients receiving mechanical ventilation, necessitating adequate monitoring and the implementation of safety measures during mobilization sessions [36]. Patient-related safety events during active mobilization, especially among intubated patients in the ICU, are subjects of significant research focus aimed at comprehending the associated risk factors [37]. While early mobilization and rehabilitation within the ICU have generally demonstrated safety, potential safety events such as hemodynamic fluctuations and desaturation may arise, underscoring the imperative of vigilant monitoring and risk assessment throughout mobilization sessions [38]. Notably, barriers to early mobilization encompass apprehensions regarding patient safety, including physiological alterations such as hypotension and hypoxemia, as well as the necessity for the removal of medical devices. These concerns mandate meticulous consideration and management to safeguard patient well-being during mobilization activities [38]. Addressing these safety concerns entails the implementation of structured protocols, interprofessional training initiatives, and fostering a culture that prioritizes patient safety. Such measures are indispensable for mitigating risks and optimizing the benefits of early mobilization therapy within the ICU setting [39].

Staffing and Resource Limitations

Implementing early mobilization therapy in the ICU needs to be improved by staffing and resource limitations, which present formidable challenges. Studies have pinpointed restricted staffing levels, time constraints, and inadequate equipment availability as primary barriers to early mobilization within the ICU [1,33,39]. Physiotherapists and healthcare professionals have voiced concerns regarding the impediments posed by constrained timeframes, heightened workloads, and staffing shortages, all of which impede their capacity to initiate early mobilization for ICU patients [1]. Moreover, the scarcity of personnel available to facilitate patient mobilization, compounded by issues such as excessive sedation, delirium, the risk of musculoskeletal injuries, and elevated workplace stress levels, exacerbates the hurdles associated with implementing early mobilization practices in the ICU [1,33,40]. Effectively surmounting these staffing and resource limitations necessitates adopting strategic measures, including workflow optimization, interprofessional training initiatives, establishing MDTs, and implementing streamlined communication processes to facilitate planning and ensure equitable distribution of mobility sessions [33]. Addressing these barriers is paramount to fostering the seamless integration of early mobilization therapy into routine clinical care within the ICU, ultimately fostering improved outcomes for critically ill patients.

Patient-Specific Considerations

Patient-specific considerations are pivotal in successfully implementing early mobilization therapy within the ICU, ensuring safety and effectiveness for critically ill patients. Factors such as physiological instability, the presence of medical devices, and the patient’s readiness are paramount in determining the appropriateness of early mobilization [1,3]. Patient safety and stability emerge as prominent concerns when addressing barriers to early mobilization in the ICU. Studies underscore the critical importance of assessing patient readiness and physiological status before mobilizing [1]. Factors such as hyperglycemia, the risk of pressure ulcers, and psychological effects necessitate vigilant monitoring during early mobilization to preempt potential complications [1].

Moreover, patient-specific barriers, including time constraints, staffing limitations, and inadequate training, can impede the initiation of early mobilization. This underscores the imperative of adopting tailored approaches based on individual patient needs and conditions [1,3]. By carefully considering these patient-specific factors, healthcare professionals can optimize the safety and efficacy of early mobilization therapy in the ICU, promoting improved patient outcomes.

Family Involvement and Support

Family involvement and support emerge as integral components of early mobilization therapy within the ICU, a notion underscored by various studies. Engaging families in mobilization endeavors has been proven safe and feasible, yielding benefits for patients and their loved ones [41-43]. Research indicates that a significant proportion of patients desire to undergo early mobilization following admission to the ICU, with the majority endorsing the view that early mobilization should be standard practice. Furthermore, many patients perceive mobilization as instrumental in facilitating recovery [42]. Moreover, family members express a keen interest in participating in mobilization activities, underscoring their willingness to actively contribute to the care of their loved ones during their ICU stay [42]. Studies further underscore the significance of family engagement, particularly within pediatric ICUs, where a positive attitude and support for early mobilization from all staff members have been shown to reassure families and foster their active participation in the rehabilitation process [43]. By incorporating families into the mobilization process, healthcare professionals can enhance patient care and contribute to a supportive environment conducive to patient recovery within the ICU.

Implementation strategies

Multidisciplinary Approach

The successful integration of early rehabilitation into routine practice within the ICU hinges on the importance of multidisciplinary collaboration. A team-based approach involving various healthcare professionals is essential to ensure patient availability, sufficient staffing, and the coordination necessary to achieve early mobilization amid the demanding ICU environment [44]. Mobility champions are pivotal in fostering a culture prioritizing early mobilization and rehabilitation within the ICU. These individuals provide leadership, communication skills, education, training, coordination, and advocacy for patient mobilization, facilitating integration into routine ICU care [9].

**Figure 2:**
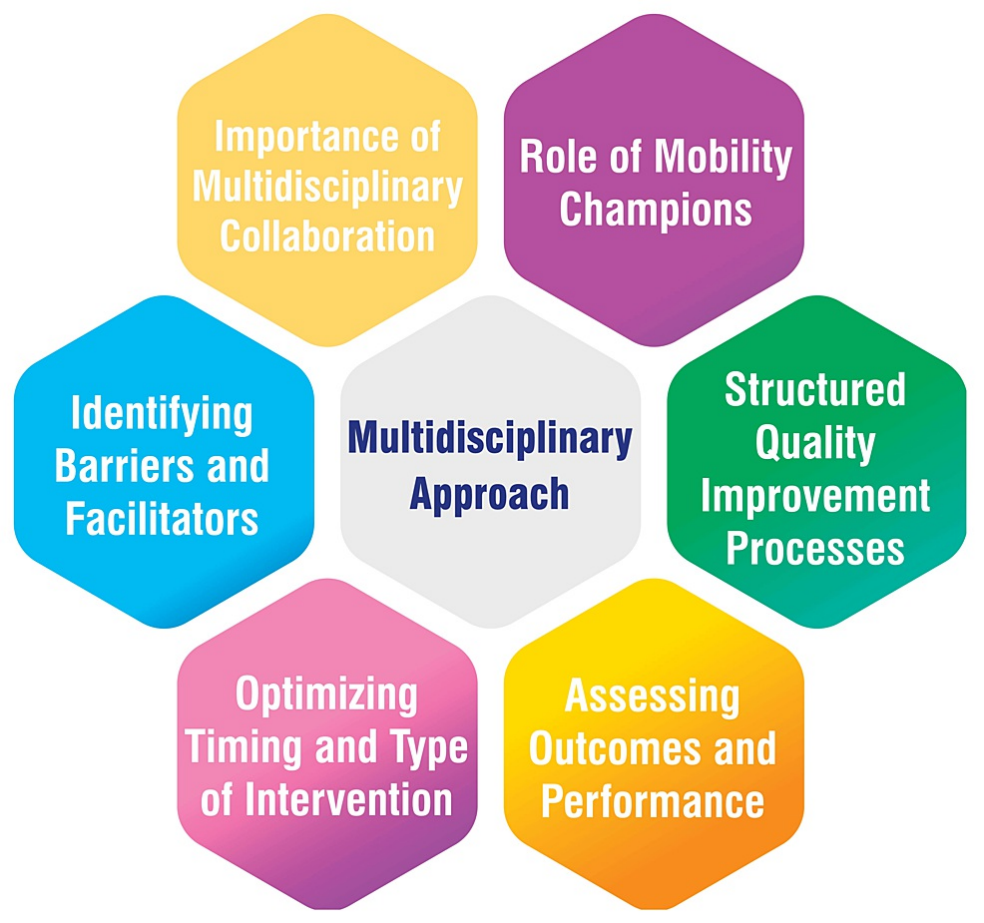
Multidisciplinary approach Image credit: Amol Singam

Structured quality improvement processes are crucial in enhancing the successful implementation of early mobilization and rehabilitation initiatives. This approach encompasses summarizing evidence, identifying barriers, establishing performance measures, and ensuring all eligible patients receive the intervention through comprehensive engagement, education, execution, and evaluation strategies [9]. Recognizing and addressing barriers to early mobilization is paramount for its effective implementation. These barriers encompass patient-related factors, structural challenges, procedural issues, and cultural considerations. Overcoming these hurdles entails implementing safety guidelines, utilizing mobility protocols, providing interprofessional training and education, and involving physician champions [9]. Evaluating interventions’ optimal timing, type, and dosage is imperative for effective early mobilization. Initiating rehabilitation shortly after ICU admission and considering various interventions such as active functional mobilization, in-bed cycle ergometry, electrical muscle stimulation, tilt tables, and other equipment can significantly enhance outcomes [9]. Developing mobility-related measures integrated into clinical care facilitates the establishment of patient goals, tracking of progress, effective allocation of resources, and evaluation of structured quality improvement programs. Understanding patients’ functioning before a critical illness is vital for tailoring interventions to meet their needs [9]. Figure 2 shows a multidisciplinary approach.

Protocol Development and Standardization

Advances in ICU care have been propelled by the development of clinical protocols and the establishment of MDTs, resulting in notable enhancements in patient management and consequent reductions in morbidity and mortality among critically ill patients [45]. Protocols play a pivotal role in the ICU, providing a structured framework for care delivery. While they do not ensure immediate improvements in care quality, protocols offer essential guidelines for attaining higher standards of care through standardization and reliance on evidence-based practices [45]. Evidence substantiates the effectiveness of protocols in ameliorating outcomes in critical care settings. Areas such as anemia management, sedation, ventilator weaning, and ventilation strategies have demonstrated improved outcomes with the implementation of protocols [45].

Despite their benefits, concerns linger regarding the potential for protocols to supplant clinical judgment, raising apprehension about compromised care quality, fostering complacency, and impeding professional learning. Critics argue that protocols may erode the decision-making process of healthcare professionals in an era marked by high technological reliance [46]. Quality improvement tools, such as checklists and structured care plans, are indispensable for standardizing care delivery within the ICU. These tools aim to enhance quality, safety, and patient satisfaction and reduce ICU length of stay by structuring care processes and ensuring adherence to best practices [47]. Successfully implementing protocols and quality improvement tools necessitates addressing local barriers, garnering clinician buy-in, and implementing ongoing strategies to maintain utilization. A comprehensive understanding of the factors conducive to successful implementation is imperative for devising effective tools to augment care delivery within the ICU [48].

Staff Education and Training

**Figure 3:**
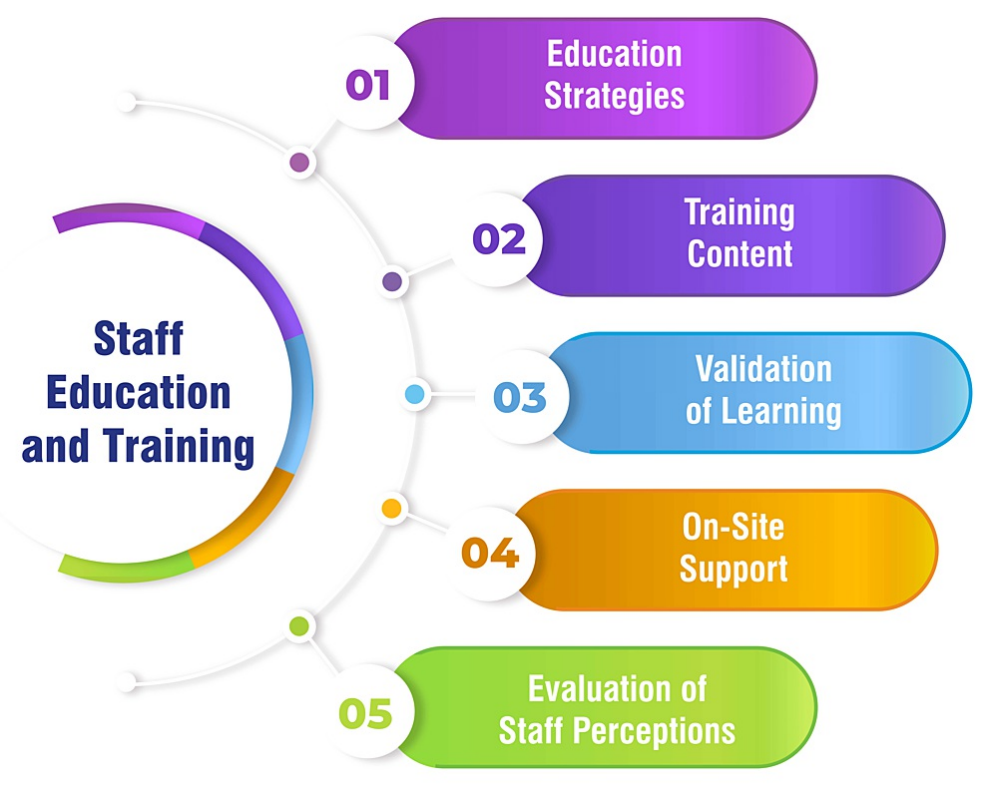
Staff education and training Image credit: Amol Singam

Various educational strategies are employed to train ICU staff on the benefits and safety of early mobilization, encompassing communication methods such as email correspondence, staff meetings, poster displays, face-to-face education sessions, and in-person training sessions tailored for an interdisciplinary cohort of volunteer champions. The educational initiatives focus on elucidating the detrimental effects of prolonged bed rest, extolling the benefits of early mobilization for ICU patients, elucidating safety protocols, and imparting knowledge on utilizing mobility protocols [33]. Staff education encompasses a range of topics, including the significance of rehabilitation in mitigating ICUAW and delirium, the safety considerations inherent in early mobility programs, and the favorable impact on patient outcomes, such as shortened ventilator duration, reduced ICU and hospital length of stay, and diminished complications. Training endeavors emphasize that critically ill patients can safely engage in movement activities [49]. The teach-back method is employed to validate staff learning during training sessions, ensuring that staff members effectively comprehend the protocols and procedures for early mobilization [33]. Following implementation, on-site support is extended to ICU departments for three weeks, facilitated by volunteers who conduct rounds through each unit, responding to inquiries, delivering real-time education to staff members, and aiding in patient mobility. This hands-on support reinforces training efforts and addresses any immediate concerns or queries from staff members [33]. Staff perceptions regarding mobility are evaluated through surveys incorporating questions on a Likert scale and soliciting free-text responses targeting existing barriers to mobility. Understanding staff perceptions is essential for addressing challenges and refining the implementation of early mobilization programs [33]. Figure 3 shows staff education and training.

Use of Technology and Assistive Devices

**Figure 4:**
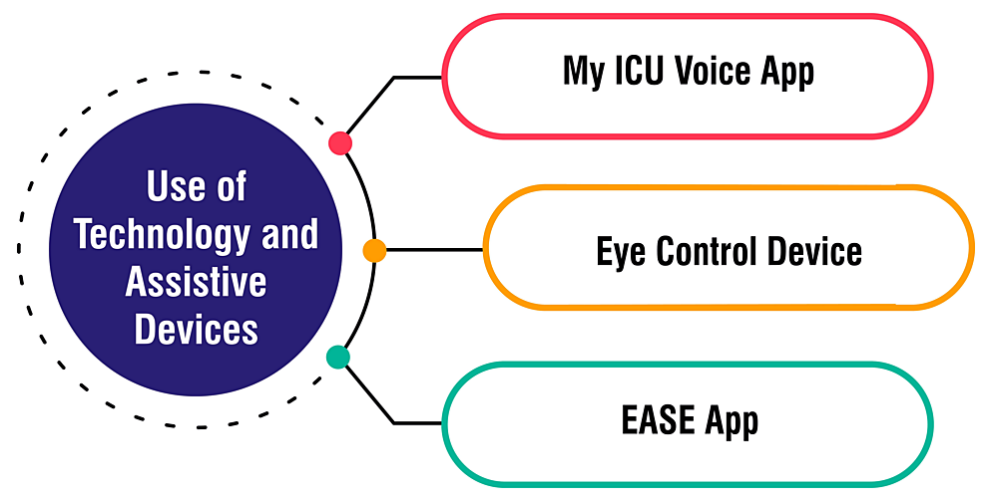
Use of technology and assistive devices Image credit: Amol Singam

The myICUvoice app, developed by an intensivist and trialed at Addenbrooke’s Hospital in Cambridge, utilizes touchscreen technology on an iPad to facilitate communication between patients and healthcare staff. This innovative tool empowers patients to express symptoms and convey their needs effectively, providing a vital communication channel that ICU staff might otherwise miss. By enabling patients to articulate their feelings and requirements, the app enhances patient-centered care within the ICU setting [50,51]. The EyeControl device, developed by an Israeli company, offers assistive communication solutions for “locked-in” patients and individuals requiring remote communication, precious during the COVID-19 pandemic. This wearable, screenless, lightweight device utilizes an infrared camera to track eye movements, translating them into audio communication. Ventilated patients can effectively communicate with medical staff and loved ones from isolated units, mitigating the risk of contagion and enhancing communication accessibility [50]. Another impactful technology in healthcare is the EASE app, which facilitates communication between healthcare providers and the families of hospitalized patients. With a simple tap, this app allows doctors or nurses to send updates, texts, photos, and videos regarding the patient’s progress. By providing real-time information and fostering seamless communication, the EASE app strengthens connections between healthcare teams and families during challenging times, enhancing the patient experience [50]. Figure 4 shows the use of technology and assistive devices.

Outcomes and implications

Functional Outcomes and Quality of Life

Functional outcomes and quality of life represent critical parameters evaluated in studies concerning early mobilization therapy within the ICU. Research findings indicate a positive correlation between early mobilization and physical therapy interventions and improved functional outcomes, encompassing factors such as muscle strength, ambulation capacity, ADLs, and mobility [27,28,52]. These interventions are tailored to address the physical dysfunction commonly observed in ICU patients, enhance functional mobility, and foster independence in daily activities [52]. Moreover, studies have underscored the beneficial impact of early mobilization on the quality of life of ICU patients, highlighting the necessity of considering not only physical outcomes but also the overall well-being and satisfaction of individuals undergoing critical care treatment [27,52]. Despite encountering challenges and barriers in implementing early mobilization protocols, the emphasis on functional outcomes and quality of life underscores the holistic approach imperative for optimizing patient recovery and long-term well-being within the ICU.

Healthcare Cost Implications

Hospitals implementing early mobility programs have reported a noteworthy reduction in direct care costs for patients, surpassing 29%, translating into substantial cost savings [53]. These programs have demonstrated their financial benefits through various avenues, including decreased ICU days, shorter hospital stays, and reduced readmission rates, contributing to overall financial advantages for healthcare institutions [53,54]. Studies have delved deeper into the financial implications, revealing that the total net present value of an early mobility program for a hospital with 1,000 yearly ICU admissions can exceed $2.3 million over seven years, underscoring the significant financial value of such initiatives [54].

An in-depth financial impact analysis highlights the annual cost-of-care savings associated with early mobility programs, encompassing reductions in ICU and non-ICU days for ventilated and non-ventilated patients and fewer days on ventilation, thereby heralding substantial financial benefits for hospitals [55]. Moreover, these initiatives have been linked to notable reductions in hospital readmission rates, resulting in additional annual savings for healthcare facilities [55].

Beyond the scope of hospitals, early mobility programs extend their value proposition to third-party payers and capitated health systems, manifesting in improved patient outcomes and reduced costs, thereby generating considerable value for these stakeholders [54]. Even in scenarios where the clinical effectiveness of an early mobility program is moderately reduced by 20%, the financial impact remains positive, thus accentuating the economic viability of investing in such programs [54].

Long-Term Effects on Morbidity and Mortality

**Figure 5:**
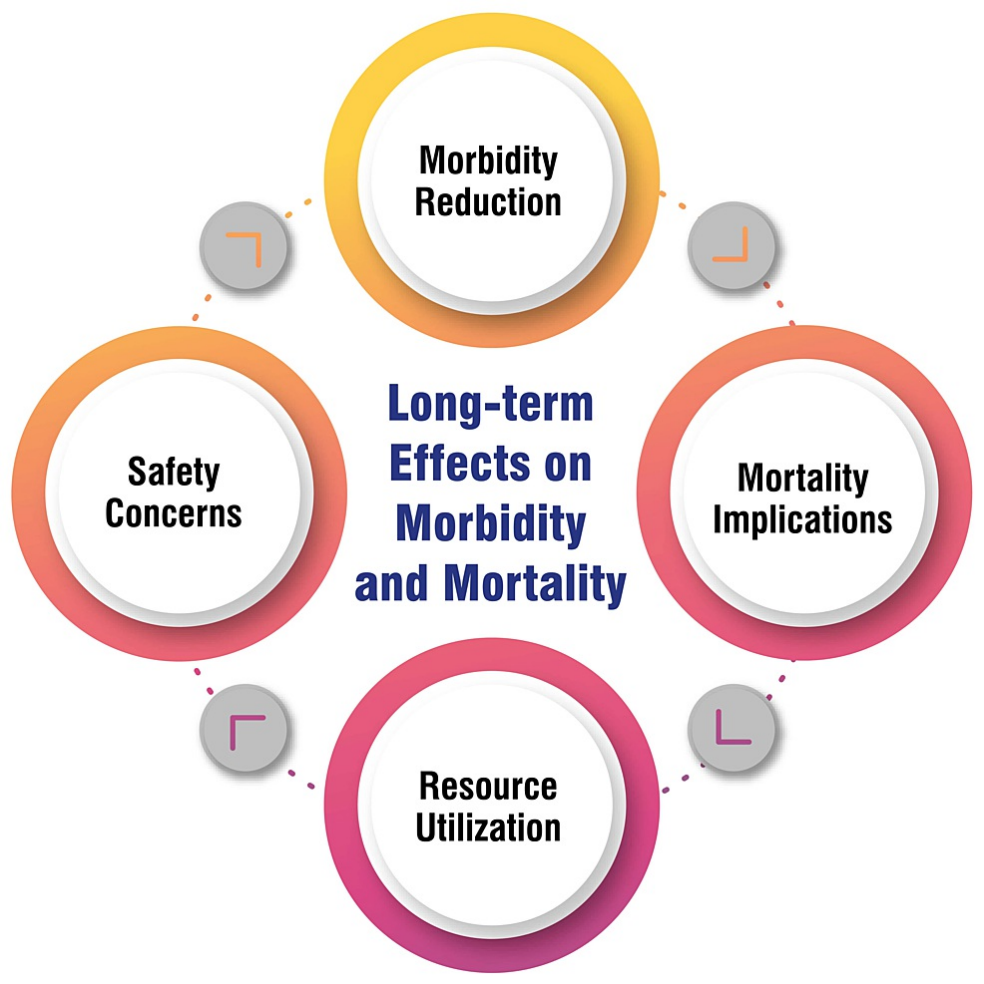
Long-term effects on morbidity and mortality Image credit: Amol Singam

Prolonged hospitalization and immobility in critically ill patients pose significant risks for long-term physical and cognitive impairments. Early mobilization therapy has emerged as a promising intervention in mitigating morbidity associated with prolonged immobilization, including muscle weakness, myopathy, and muscular atrophy [29]. While early mobilization demonstrates short-term benefits, such as reduced mechanical ventilation days and hospital length of stay, its implications on long-term mortality remain under scrutiny. Studies suggest that active mobilization and rehabilitation in the ICU do not increase short- or long-term mortality rates. Instead, they may contribute to improvements in muscle strength, mobility status, and participation restriction outcomes [56]. Implementing early mobilization interventions in the ICU may only sometimes result in direct cost savings due to the intricacies involved. The resource and labor burdens associated with early mobilization programs necessitate careful evaluation against potential benefits to ensure efficient resource allocation [29]. Safety concerns, particularly in cardiac surgery patients, have been linked to adverse events during early mobilization, including significant hemodynamic alterations. Addressing these safety concerns and optimizing protocols for safe mobilization practices is imperative to maximize the benefits of early mobilization while minimizing associated risks [29]. Figure 5 shows the long-term effects on morbidity and mortality.

Future directions and research needs

Areas for Further Investigation

**Figure 6:**
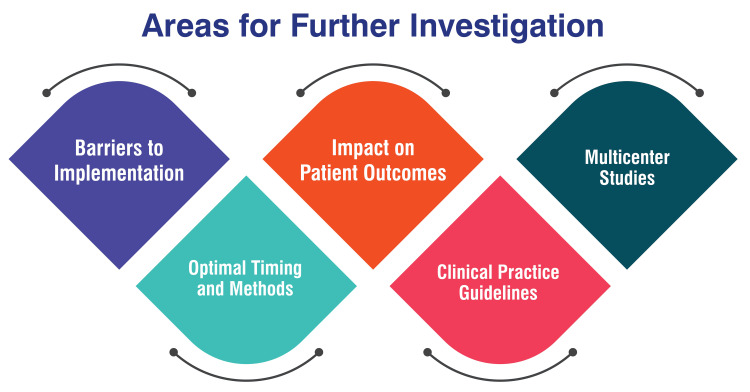
Areas for further investigation Image credit: Amol Singam

Further research is imperative to identify and address barriers hindering the implementation of early mobilization in the ICU. These barriers, including deep sedation, lack of coordination with rehabilitation professionals, and limited understanding of the benefits of early rehabilitation, need a thorough investigation to facilitate effective strategies for overcoming them [52]. Investigating the optimal timing and methods for early mobilization in critically ill patients is paramount. Understanding the appropriate initiation time for mobilization and determining the most effective techniques can significantly improve patient outcomes and prevent delays in achieving early mobilization milestones [52]. Conducting multicenter studies is essential to reduce bias and explore day-to-day changes in barriers to implementing early mobilization. These studies offer valuable insights into the challenges encountered across diverse ICU settings, thereby informing the development of comprehensive rehabilitation strategies [52]. Research endeavors should prioritize assessing the impact of early mobilization on various patient outcomes. These outcomes encompass independence in ADLs, quality of life, duration of mechanical ventilation, ICU and hospital length of stay, and overall physical function post-discharge. A thorough evaluation of these metrics can provide comprehensive insights into the efficacy of early mobilization interventions [52]. Continuous evaluation and refinement of clinical practice guidelines for early mobilization in the ICU are indispensable. Addressing issues such as the optimal dose of mobilization, criteria for patient selection, and timing of early mobilization can further optimize the implementation of early mobilization protocols, ensuring standardized and effective care delivery [57]. Figure 6 shows areas for further investigation.

Innovations in Early Mobilization Techniques

**Figure 7:**
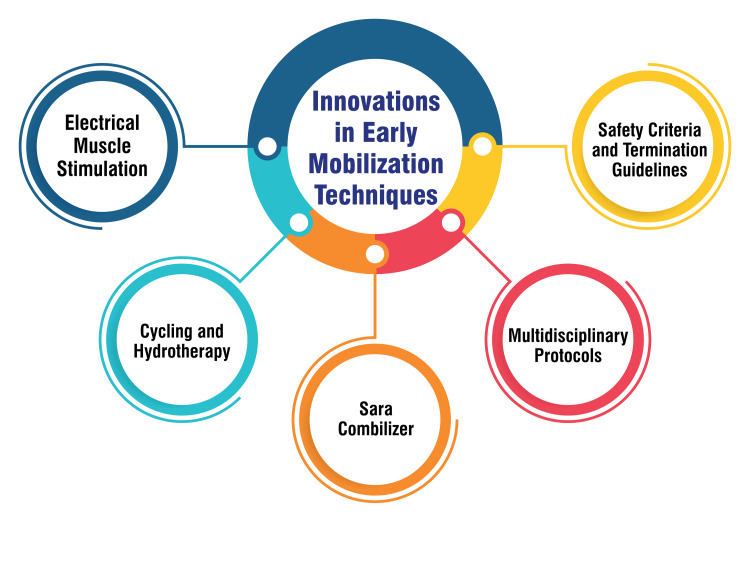
Innovations in early mobilization techniques Image credit: Amol Singam

Newer techniques, such as electrical muscle stimulation, have emerged as safe and effective modalities for facilitating early mobilization and improving outcomes for critically ill patients in the ICU [3]. Innovative approaches like cycling and hydrotherapy have demonstrated positive outcomes and safety in early mobilization endeavors, contributing significantly to the evolution of rehabilitation practices within the ICU [3]. The Sara Combilizer, a device highlighted in the sources, represents a notable advancement in aiding patient mobility in the ICU while maintaining safety standards [3]. Integrating multidisciplinary protocols incorporating early mobility alongside interventions like awakening/breathing coordination and delirium monitoring has been acknowledged as a forward-thinking strategy to bolster patient outcomes and foster early mobilization in the ICU [58]. Several studies have delineated safety criteria and termination guidelines for both in-bed and out-of-bed mobilization, offering a structured approach to uphold patient safety during early mobilization initiatives [3]. Figure 7 shows innovations in early mobilization techniques.

Integration of Early Mobilization Into Standard ICU Care

**Figure 8:**
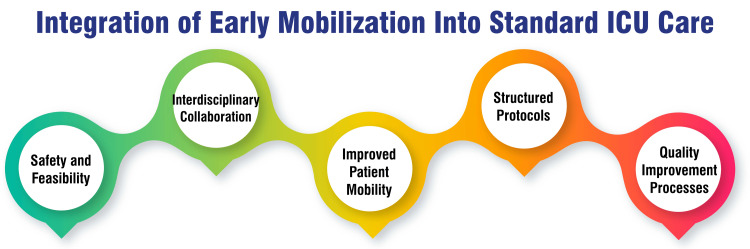
Integration of early mobilization into standard ICU care Image credit: Amol Singam

Studies emphasize the safety and feasibility of early physical therapy and ICU mobilization for critically ill patients, demonstrating their potential to enhance the attainment of mobility milestones within the ICU setting [1]. Interdisciplinary collaboration plays a pivotal role in successfully integrating early mobility protocols into ICU care, necessitating the involvement of various healthcare professionals, such as physicians, nursing staff, respiratory therapists, physical therapists, and occupational therapists [33]. Implementing early mobility protocols has been shown to improve patient mobility within the ICU, as evidenced by decreased time from admission to ambulation and an increased proportion of patients engaging in ambulation while in the ICU [33]. Developing and implementing structured protocols for early mobility are crucial components for successful integration into standard ICU care. These protocols should prioritize interdisciplinary collaboration, streamline workflow, and optimize coordination of tasks to ensure effectiveness [33]. Utilizing structured quality improvement processes is essential for enhancing the successful implementation of early mobilization and rehabilitation in the ICU. These processes ensure that all eligible patients receive the intervention and address barriers such as sedation, a lack of equipment, and coordination issues [9]. Figure 8 shows the integration of early mobilization into standard ICU care.

## Conclusions

This review highlights the critical role of early mobilization therapy in improving outcomes for ICU patients, emphasizing benefits like reduced muscle weakness and shorter stays in both the ICU and hospital settings. Despite its advantages, implementing early mobilization requires a multidisciplinary effort, standardized protocols for safety and efficacy, and overcoming challenges such as staffing limitations and patient safety concerns. To maximize patient recovery and quality of life, ongoing research and the development of clear, standardized mobilization guidelines are essential for widespread adoption in critical care.
